# Characterisation of new anti-O157 bacteriophages of bovine origin representing three genera

**DOI:** 10.1007/s00203-022-02839-4

**Published:** 2022-03-30

**Authors:** Domonkos Sváb, Linda Falgenhauer, Viktória Papp, Manfred Rohde, Trinad Chakraborty, István Tóth

**Affiliations:** 1 Veterinary Medical Research Institute, Hungária krt. 21., 1143 Budapest, Hungary; 2 Institute of Hygiene and Environmental Medicine and German Center for Infection Research (DZIF), Partner Site Giessen-Marburg-Langen, Justus Liebig University Giessen, Schubertstrasse 81, 35392 Giessen, Germany; 3 Helmholtz Centre for Infection Research, Inhoffenstrasse 7, 38124 Braunschweig, Germany; 4 Institute of Medical Microbiology, and German Center for Infection Research (DZIF), Partner Site Giessen-Marburg-Langen, Justus Liebig University Giessen, Schubertstrasse 81, 35392 Giessen, Germany

**Keywords:** *Escherichia coli* O157, Bacteriophage, Biocontrol, *Vequintavirus*, *Tequatrovirus*, *Dhillonvirus*

## Abstract

Shiga-toxin-producing *Escherichia coli* (STEC) strains of the serogroup O157 are foodborne pathogens associated with severe clinical disease. As antibiotics are counter-indicated for treatment of these infections, they represent prime candidates for targeted application of bacteriophages to reduce infection burden. In this study, we characterised lytic bacteriophages representing three phage genera for activity against *E. coli* O157 strains. The phages vb_EcoM_bov9_1 (*Tequatrovirus*), vb_EcoM_bov11CS3 (*Vequintavirus*), and vb_EcoS_bov25_1D (*Dhillonvirus*) showed effective lysis of enterohaemorrhagic *E. coli* EHEC O157:H7 strains, while also exhibiting activity against other strains of the O157 serogroup, as well as of the ‘big six’ (STEC) serogroups, albeit with lower efficiency. They had a burst size of 293, 127 and 18 per cell and a latent period of 35, 5 and 30 min, respectively. In situ challenge experiments using the O157 Sakai strain on minced beef showed a reduction by 2–3-fold when treated with phages at a 0.1 MOI (multiplicity of infection), and approximately 1 log reduction when exposed to MOI values of 10 and 100. A cocktail of the phages, applied at 10 × and 100 × MOI showed 2 to 3 log reduction when samples were treated at room temperature, and all treatments at 37 °C with 100 × MOI resulted in a 5 to 6 log reduction in cell count. Our results indicate that the phages vb_EcoM_bov9_1 and vb_EcoM_bov11CS3, which have higher burst sizes, are promising candidates for biocontrol experiments aimed at the eradication of *E. coli* O157 strains in animals or foodstuff.

## Introduction

Shiga toxin-producing (STEC) and enterohaemorrhagic (EHEC) *Escherichia coli* of the O157 serogroup are foodborne zoonotic pathogens considered a serious threat to public health, with high number of hospitalisations and a significant annual mortality rate in the developed world (reviewed by Caprioli et al (2005) and Kim et al. (2020). They are capable of causing haemorrhagic colitis (HC) and the life-threatening haemolytic-uraemic syndrome (HUS; reviewed by Caprioli et al. 2005) and have a very low infective dose (< 100 cells, reviewed by Todd et al. 2008). Their main natural reservoir is healthy cattle (Terajima et al. 2017). As its main virulence factors, the Shiga toxins (Stx) are prophage-encoded, antibiotic treatment of infection is generally counter-indicated, as it can lead to an increase in toxin production (Tarr et al. 2005). In addition, the emergence of antibiotic-resistant strains of this pathotype has being reported in recent years (Mir and Kudva 2019; Walusansa et al. 2020).

Recently the use of bacteriophages has been given increased consideration as antibacterial agents (reviewed by Kortright et al. 2019), with *E. coli* O157 strains being frequent candidates for the detection of phages and development of targeted phage products. Moreover, a few products are already commercialised and approved for application (reviewed by Wang et al. 2017). As EHEC are foodborne enteric pathogens, the main approach of bacteriophage experiments in this case is preventive biocontrol in foodstuff, although attempts were also made to eradicate them from live animals (Rivas et al. 2010; Raya et al. 2011). Several phages have been identified as potentially suitable for biocontrol against O157 strains. Various experiments have been performed with phages applied against O157 strains in different foodstuffs ranging from beef (Hudson et al. 2015) to vegetables (Sharma et al. 2009; Patel et al. 2011; Hong et al. 2014) with promising results.

Our hypothesis was that lytic phages effective against O157 serogroup strains can be isolated from healthy cattle, their natural reservoir. Besides the discovery of potentially useful phages, characterisation of phages co-existing with these bacteria is important in understanding the ecology of the pathogens within their reservoir-hosts.

Previously, we determined the genome sequences of eleven bacteriophages of bovine origin (Sváb et al. 2021). In the present study, we focused on the detailed characterisation of representatives of the three phage genera which they belong to (*Tequatrovirus* or T4-like, *Vequintavirus* or rV5-like and *Dhillonvirus* or HK578-like phages), with the purpose of assessing their potential as biocontrol agents.

## Materials and methods

### Bacteriophage isolation

Isolation of bacteriophages was described in Sváb et al (2021). The summary of bacteriophages found in the study, with those included in the phenotypic characterisation, are listed in Table 1.

**Table 1:** List of bacteriophages isolated and sequenced by Sváb et al (2021)

Phage	Source	Genome length (nt)	Predicted genus	Accession number
**vb_EcoM_bov9_1 (phage 9)**	Bovine faecal sample	166,440	*Tequatrovirus*	MT884006
vb_EcoM_bov10K1	Cattle farm environment	166,441	*Tequatrovirus*	MT884007
vb_EcoM_bov10K2	Cattle farm environment	135,960	*Vequintavirus*	MT884008
**vb_EcoM_bov11CS3 (phage 11)**	Bovine faecal sample	135,960	*Vequintavirus*	MT884009
vb_EcoM_bov22_2	Bovine faecal sample	135,961	*Vequintavirus*	MT884010
vb_EcoM_bov25_3	Bovine faecal sample	135,961	*Vequintavirus*	MT884011
vb_EcoS_bov11C2	Bovine faecal sample	44,612	*Dhillonvirus*	MT884012
vb_EcoS_bov16_1	Bovine faecal sample	44,745	*Dhillonvirus*	MT884013
vb_EcoS_bov22_1	Bovine faecal sample	44,612	*Dhillonvirus*	MT884014
**vb_EcoS_bov25_1D (phage 25)**	Bovine faecal sample	44,747	*Dhillonvirus*	MT884015
vB_EcoS_bov15_1	Bovine faecal sample	44,700	*Dhillonvirus*	MT951623

Phages used in the detailed phenotypic characterisations of the current study are bolded

### Host spectrum and efficiency of plating (EOP)

The host spectrum and efficiency of plating (EOP) of three chosen phages, representing the three genera, was assessed, namely those of vB_EcoM_bov9_1, vB_EcoM_bov11CS3 and vB_EcoS_bov25_1D (representing T4-like, rV5-like and HK578-like phages, respectively). We will refer to these phages heretofore as phage 9, 11 and 25, respectively. Bacterial strains used in these experiments are listed in Table 2. Strains representing the ‘big six’ serogroups of STEC (Brooks et al. 2005; Bertoldi et al. 2017) were kindly provided by Tünde Mag (National Public Health Center, Budapest, Hungary). The presence of key virulence genes, *stx1, stx2* (encoding the two types of Shiga toxin) and *eae* (encoding the adhesin intimin) was checked by PCR systems according to Scheutz et al (2012) and China et al (2000), respectively. Layered soft agar plating and spot assays were performed according to Strauch et al. (2001).

**Table 2:** Efficiency of plating (EOP) of phages vB_EcoM_bov9_1 (phage 9), vB_EcoM_bov11CS3 (phage 11) and vB_EcoS_bov25_1D (phage 25) on *E. coli* O157 and ‘big six’ strains with the titre on EHEC O157:H7 Sakai used as reference written in bold

Strain	Pathotype	Phage type	Serotype	EOP vb_Ecom_bov9_1	EOP vb_EcoM_bov11CS3	EOP vb_EcoS_bov25_1D	Strain reference
**Sakai**	**EHEC**	**14**	**O157:H7**	**1 (ref.)**	**1 (ref.)**	**1 (ref.)**	Hayashi (2001)
EDL933	EHEC	21	O157:H7	0.71	10^–8^ > n > 10^–9^	0.4	Perna et al. (2001)
34	EHEC	21	O157:H7	10^–6^ > *n* > 10^–7^	0	0	Tóth et al. (2009)
52	EHEC	33	O157:H7	4.28	1.25 × 10^–10^	10^–4^ > *n* > 10^–5^	Tóth et al. (2009)
318	EHEC	8	O157:H7	10^–6^ > *n* > 10^–7^	10^–8^ > *n* > 10^–9^	0	Tóth et al. (2009)
64	EPEC	8	O157:H7	1.43	10^–9^ > *n* > 10^–10^	10^–4^ > *n* > 10^–5^	Tóth et al. (2009)
65	EPEC	33	O157:H7	1.43	5 × 10^–4^	0.01	Tóth et al. (2009)
B20	AT^a^	NT-R^b^	O157:H12	10^–9^ > *n* > 10^–10^	10^–8^ > n > 10^–9^	0	Tóth et al. (2009)
T22	AT^a^	NC^c^	O157:H43	10^–5^ > *n* > 10^–6^	0.225	10^–3^ > n > 10^–4^	Tóth et al. (2009)
HNCMB30041	EHEC	N/A	O26:H11	10^–5^ > *n* > 10^–6^	10^–5^ > n > 10^–6^	10^–4^ > *n* > 10^–5^	This study
HNCMB30059	STEC	N/A	O45:H10	0	0	0	This study
HNCMB30113	STEC	N/A	O103:H8	1.75 × 10^–1^	6.67 × 10^–1^	7 × 10^–1^	This study
HNCMB30121	EHEC	N/A	O111:NM	10^–5^ > *n* > 10^–6^	10^–3^ > *n* > 10^–4^	10^–3^ > *n* > 10^–4^	This study
HNCMB30131	STEC	N/A	O121:H10	5 × 10^–2^	1.33 × 10^–1^	1.5 × 10^–1^	This study
HNCMB30154	EPEC	N/A	O145:NM	0	0	0	This study

^a^Atypical

^b^Non-typeable and phage resistant

^c^Non-characteristic

### Phage and phage DNA purification

Genomic DNA isolation and sequencing methods and accession numbers of the genomes were published in Sváb et al. (2021) and are listed in Table 1. Phages with the same numeric designation originated from the same samples, and despite the purification of single plaques, sequencing showed that some of the samples were mixed. Further purification of phages was performed by selecting single plaques and testing them using PCR systems designed with PrimerBLAST on the NCBI website, specific for marker genes unique to each genus.

Phage DNA for PCR was isolated by first treating the high titre (> 10^9^ PFU/ml) phage suspensions with DNAse I solution (Sigma-Aldrich) according to the manufacturer’s instruction to eliminate residual bacterial DNA, then boiling the suspension to extract DNA from intact phage particles. For the PCR, DreamTaq Green Mastermix (ThermoScientific) was used according to the manufacturer’s instruction, with the primers listed in Table 3. High titre (> 10^9^ PFU/ml) phage stocks positive for only one marker gene were considered to contain a single phage, and these were used in the phenotypic experiments.

**Table 3:** List of primers designed in this study and used in PCRs for separating phage stocks

Primer name	Sequence 5ʹ- > 3ʹ	Position in reference genome	Gene encoded in amplified region	Reference phage genome	Reference GenBank accession number
Dhillon_cs_f	AGTCCATGAGCAACAAGGCA	3688–3704	Capsid and scaffold protein	vb_EcoS_bov25_1D	MT884015.2
Dhillon_cs_r	CGCTTCCCTTTCGTATCGGG	4263–4282	Capsid and scaffold protein	vb_EcoS_bov25_1D	MT884015.2
T4_bp_f	GGAGACTATCCGAAGACTTGGC	112,577–112,598	Baseplate	vb_EcoM_bov9_1	MT884006.2
T4_bp_r	CGCTCGGCAGGAATCATTTT	113,105–113,124	Baseplate	vb_EcoM_bov9_1	MT884006.2
rV5_rib_f	GACCAGTGCACGATCTCTGT	116,823–116,842	Ribonucleotide reductase of class III (anaerobic), large subunit	vb_EcoM_bov11CS3	MT884009.2
rV5_rib_r	ACTGACATGGGCGTTACCAA	117,235–117,254	Ribonucleotide reductase of class III (anaerobic), large subunit	vb_EcoM_bov11CS3	MT884009.2

### Phylogenetic investigation

All pairwise comparisons of the nucleotide sequences were conducted using the Genome-BLAST Distance Phylogeny (GBDP) method (VICTOR, (Meier-Kolthoff and Göker 2017)) under settings recommended for prokaryotic viruses. The resulting intergenomic distances were used to infer a balanced minimum evolution tree with branch support via FASTME including SPR post processing (Lefort et al. 2015) for the D0 distance formula recommended for nucleotide sequences of prokaryotic viruses. Branch support was inferred from 100 pseudo-bootstrap replicates each. Trees were rooted at the midpoint (Farris 1972) and visualized with FigTree (Rambaut 2006). Taxon boundaries at the species, genus and family level were estimated with the OPTSIL program (Göker et al. 2009), the recommended clustering thresholds (Meier-Kolthoff and Göker 2017) and an F value (fraction of links required for cluster fusion) of 0.5 (Meier-Kolthoff et al. 2014).

For each genus, whole bacteriophage genomes designated in GenBank to be members of the same genus were used. For the *Tequatrovirus* genus, species listed in the genus on the ICTV website (Lavigne and Ceyssens 2011) were used where whole genome sequences were available. For the *Vequintavirus* and *Dhillonvirus* genera, MEGABLAST hits in GenBank showing > 95% coverage and/or nucleotide similarity were included.

### Transmission electron microscopy

Morphological investigation of phages was performed with transmission electron microscopy (TEM). Five µl drops of high titre (> 10^9^ PFU/ml) bacteriophage suspensions were placed on parafilm, absorbed onto carbon film, washed in TE buffer (10 mM TRIS, 1 mM Na_2_EDTA, pH 6.9) and negatively-stained with 2% aqueous uranyl acetate, pH 5.0. Carbon film was collected with 300 mesh copper grids and excess negative-stain was removed with filter paper and subsequently air-dried. Samples were examined in a TEM 910 transmission electron microscope (Carl Zeiss, Oberkochen) at an acceleration voltage of 80 kV. Images were recorded digitally at calibrated magnifications with a Slow-Scan CCD-Camera (ProScan, 1024 × 1024, Scheuring, Germany) with ITEM-Software (Olympus Soft Imaging Solutions, Münster, Germany). Contrast and brightness were adjusted with Adobe Photoshop CS3.

### One-step growth experiment

To determine the burst size and latent period of the phages, one-step growth experiments were performed according to Bassiri (Bassiri) and Sváb et al (2018a). Briefly, 2 × 10^8^ cells of the EHEC O157:H7 Sakai strain were mixed with 2 × 10^6^ particles of the respective phage, setting MOI to 0.01 in Luria–Bertani broth (LB) and incubated for 20 min at room temperature. After incubation the mixture was centrifuged at 6000 × g for 10 min, the pellet was resuspended in 50 ml of fresh LB and incubated at 37 °C with shaking at 180 rpm for 1 h, and in the case of phage 25, for 1.5 h. Samples were taken every 5 min, or 10 min for phage 25 and plated on layered soft agar for counting. Three independent experiments were run in two parallels. Burst size was determined as a ratio of the phage count before and after the burst.

### In situ growth reduction experiment

In situ biocontrol potential of phages 9, 11 and 25 was investigated according to Sváb et al. (2018b), with some modifications. One gram of minced beef, bought at a local supermarket was inoculated with high numbers (10^7^ or 10^8^) of cells of a derivative of *E. coli* O157:H7 Sakai strain resistant to rifampicin and nalidixic acid, grown in LB broth. This double resistant mutant of the Sakai strain was obtained by first spreading 1 ml of overnight culture of the verified rifampicin resistant mutant, previously obtained as described by Sváb et al. (2018b), on LB agar plates containing 100 μg/ml of rifampicin and 50 μg/ml of nalidixic acid. Surviving colonies after two selective passages were used in the experiments. After inoculation of the beef samples, particles of the respective phages suspended in LB broth were added in adequate quantities to achieve multiplicity of infection (MOI) values 0.1, 10 and 100. The samples were mixed with brief but vigorous shaking.

Phages were applied either alone or in a mixture, a ‘cocktail’ containing equal amounts of each of the three. Control tubes with bacterial cells alone were also used. The experiment was conducted in triplicates with all incubating conditions and MOI values. Three types of incubation were used: 2 h at 37 °C, 24 h at 25 °C and 48 h at 4 °C. After incubation, samples were homogenized and serial dilutions were made, which were plated on LB agar plate containing 100 μg/ml of rifampicin and 50 μg/ml of nalidixic acid. Number of surviving bacterial colonies was determined after 16 h incubation at 37 °C. Preliminary tests were conducted in order to exclude the presence of bacteria resistant to rifampicin and nalidixic acid in the meat, as well as that of phages showing lytic activity on the propagating strain. Tests with phage-free controls and treated samples were also conducted to determine dilutions that yield easily countable numbers of colonies.

## Results

### Separated single representative phages

Even though genomes of the phages used in the current study were available (Table 1), to ensure the validity of the phenotypic characterisation, separation of mixed samples had to be carried out. After repeatedly taking single plaques and confirming the presence of single phages by marker gene-specific PCR designed for this study, clean and high titre stocks (> 10^9^ PFU/ml) of three phages designated phage 9, 11 and 25 (original designations: vB_EcoM_bov9_1, vB_EcoM_bov11CS3 and vB_EcoS_bov25_1D), representing the *Tequatrovirus, Vequintavirus* and *Dhillonvirus* genera respectively, were used in the characterisation experiments.

### Host spectrum and EOP

The host spectrum and EOP of the phages was tested on *E. coli* strains representing EHEC, EPEC and atypical pathotypes of the O157 serogroup with the prototypic EHEC O157:H7 strain Sakai used as reference. Phage 9 was the only one which showed effective lysis of bovine EHEC and EPEC strains together with the prototypic human EHEC strains. Phage 11 only exhibited mild lytic activity for all strains. There were three strains on which phage 25 did not show lysis, and it was only mildly lytic for all the other strains apart from the reference strain. Out of representative strains of the ‘big six’ serogroups, the O45 and O145 strains proved to be completely resistant to all three phages, while the O103 and O121 strains were lysed effectively by all of them. The detailed results are shown in Table 2.

### Phylogenetic relations

**Fig. 1:**
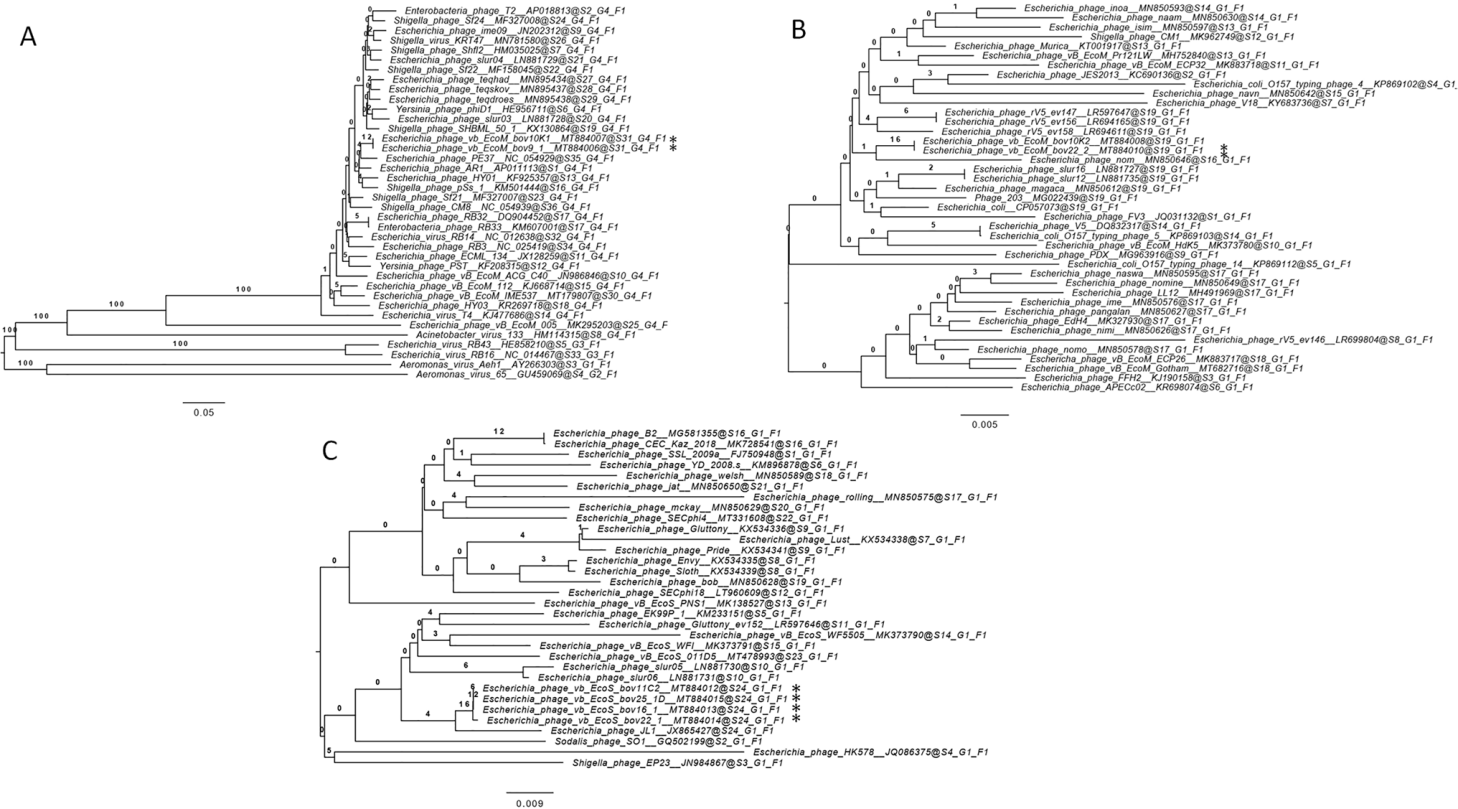
Genome-BLAST distance phylogeny trees of bacteriophage genomes belonging to the *Tequatrovirus* (**A**), *Vequintavirus* (**B**) and *Dhillonvirus* (**C**) genuses made with VICTOR (Meier-Kolthoff and Göker 2017) using the D0 distance formula recommended for prokaryotic viruses. Representatives of the phage genome set described by Sváb et al. (2021) are marked with asterisks on each tree

A whole genome-based phylogenetic tree of the phages representing each genus is shown in Fig. 1. Within each genus, all new phages sequenced in the previous study (Sváb et al. 2021) grouped together, pointing to their close relationship. While they also bear close similarities to phages in the same genus, they form distinct, new clusters within each genus.

### Phage morphology

The morphologies of phages corresponded to the families to which their genera, determined by sequence similarity, belonged to. Phage 9 displayed the characteristic *Myoviridae* morphology with a contractile tail of 115.2 ± 4.1 nm length and an icosahedral head with a size of 86.6 ± 4 by 109.4 ± 15.2 nm.

Phage 11 showed similar morphology with a contractile tail of 117.1 ± 4.5 nm in length and a head size of 83.4 ± 3.8 by 93.6 ± 2.1 nm.

**Fig. 2:**
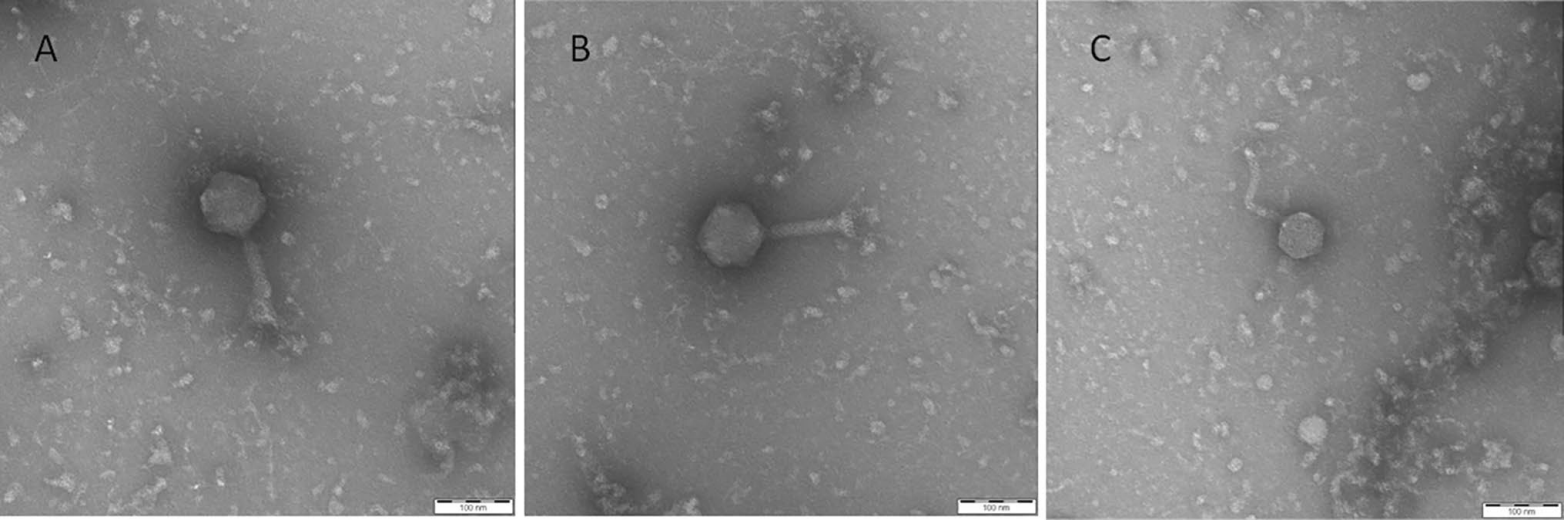
Transmission electron micrographs of phages vB_EcoM_bov9_1 (**A**), vB_EcoM_bov11CS3 (**B**) and vB_EcoS_bov25_1D (**C**)

Phage 25 showed the *Siphoviridae* morphology with a flexible 136.5 ± 11.7 nm long tail and an icosahedral head with a size of 61.2 ± 1.3 by 65.8 ± 2.2 nm (Fig. 2).

### Burst size and latent period

**Fig. 3:**
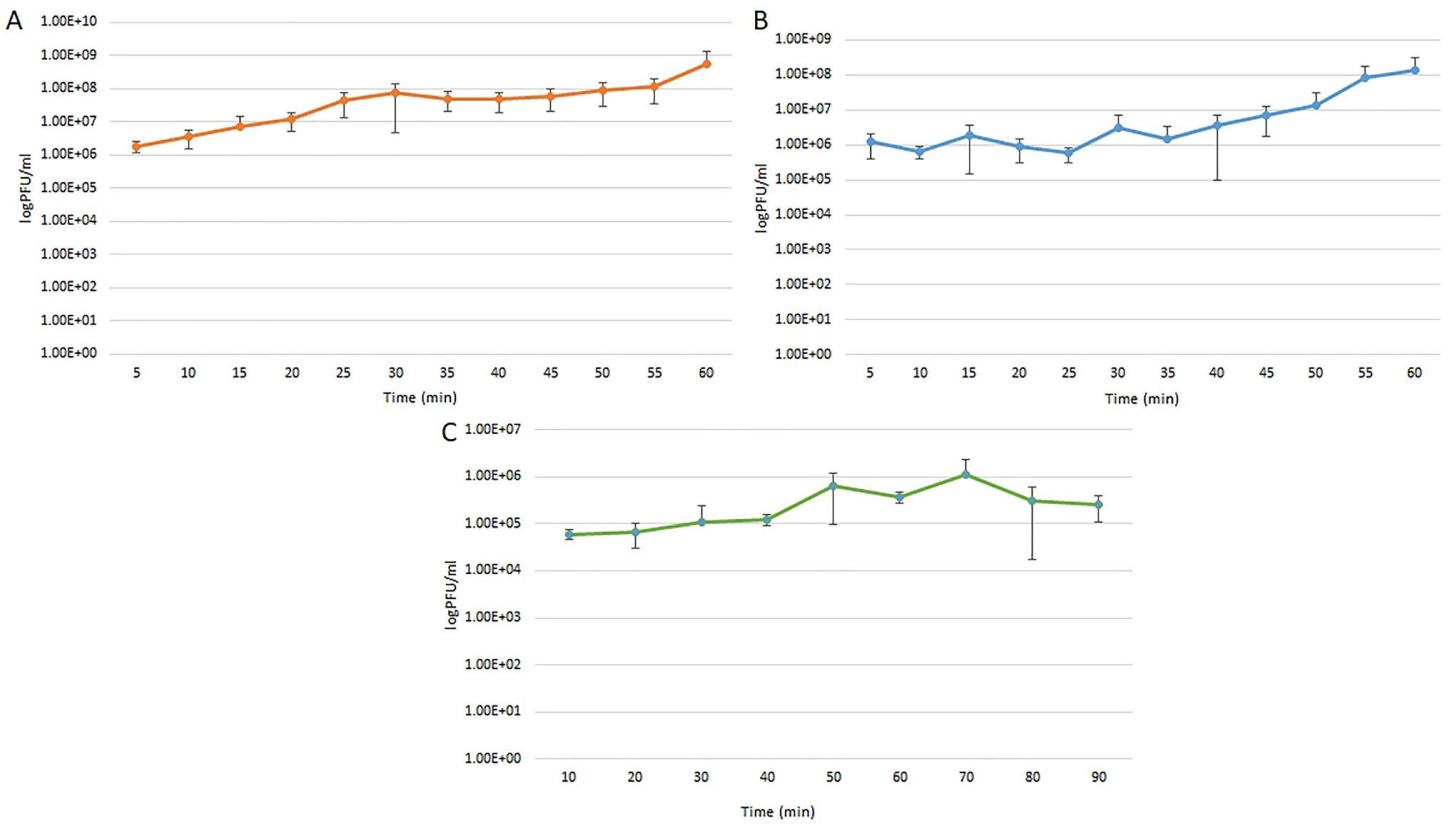
One-step growth curves of phages vB_EcoM_bov9_1 (**A**), vB_EcoM_bov11CS3 (**B**) and vB_EcoS_bov25_1D (**C**)

The burst sizes of phages 9, 11 and 25 were 293, 127 and 18, respectively; while their latent periods were 5, 30 and 35 min, respectively. The one-step growth curve of each phage is shown in Fig. 3.

### In situ growth reduction

The average growth reduction measured in the treated beef samples is shown in Table 4. In general, the colony forming unit reduction of about one order of magnitude was observable with all treatments with the MOI of 0.1 and 10. The MOI of 10 was generally 3 to 4 times more effective than the MOI of 0.1. The MOI of 100 proved by far the most effective treatment, as it reduced the CFU by 3 orders of magnitude when the phages were applied as a cocktail at the 24 h, 25 °C, and by 5 to 6 orders of magnitude after a 2 h, 37 °C treatment.

**Table 4:** Growth reduction of the rifampicin and nalidixic acid resistant mutant of EHEC O157:H7 Sakai strain in minced beef with various bacteriophage treatments

Average CFU reduction									
MOI	0.1			10			100		
Phage treatment	48 h @ 4 °C	24 h @ 25 °C	2 h @ 37 °C	48 h @ 4 °C	24 h @ 25 °C	2 h @ 37 °C	48 h @ 4 °C	24 h @ 25 °C	2 h @ 37 °C
vb_EcoM_bov9_1	2.40	4.12	1.43	16.73	34.40	7.24	6.47	407.19	7.22 × 10^5^
vb_EcoM_bov11CS3	1.38	3.41	1.20	1.00	13.14	6.87	58.57	35.22	6.5 × 10^6^
vb_EcoS_bov25_1D	2.17	4.49	1.37	0.63	7.58	13.10	3.04	11.02	2.13 × 10^6^
Cocktail	1.90	1.94	3.26	9.26	341.00	7.63	5.12	4964.38	1.86 × 10^6^

CFU reduction is given as the CFU of the phage-free control sample divided by the CFU of the treated samples after incubation. All values are an average of three experiments

## Discussion

We performed detailed phenotypic characterisation of three previously sequenced bovine coliphages capable of lysing *E. coli* O157 strains that are representatives of three phage genera (Sváb et al. 2021) and assessed their phylogenetic relations to members of the respective genera (Fig. 1).

Phages 9 (vb_EcoM_bov9_1) and vb_EcoM_bov10K1 are members of the *Tequatrovirus* genus, known as T4-like phages. These myoviruses have large genomes and have been investigated for decades in phage biology (Miller et al. 2003). Since the renewed interest in phage therapy and biocontrol, several T4-like phages have been described showing lytic activity on *E. coli* O157 strains (Sharma et al. 2009; Raya et al. 2011; Carter et al. 2012).

Phages vb_EcoM_bov10K2, vb_EcoM_bov11CS3 (Phage 11), vb_EcoM_bov22_2, and vb_EcoM_bov25_3 are of the *Vequintavirus* genus, or rV5-like phages which are a recently characterised group of phages, with a relatively large genome and broad host range, found to be active on *E. coli* O157 strains among others (Santos et al. 2011; Kropinski et al. 2013; Sváb et al. 2018b).

Of the recently described *Dhillonvirus* genus (Li et al. 2010, 2019) only one phage has been reported to be active against *E. coli* O157:H7 strains (Pan et al. 2013). In this study, five new members of this genus, vb_EcoS_bov11C2, vb_EcoS_bov15_1, vb_EcoS_bov16_1, vb_EcoS_bov22_1, and vb_EcoS_bov25_1D (phage 25), all active on the prototypic EHEC O157:H7 Sakai strain, were isolated. Members of this genus have a uniform genome sized between 43.7 and 45.2 kb with a very conserved structure (> 90% nucleotide-level overall similarity; Li et al. 2019).

Regarding host spectrum, phage 9, representing the T4-like phages lysed the broadest range of strains, showing lytic activity on all O157 strains tested. Phage 11 (rV5-like) produced lysis on all the other strains (Table 2). Phage 25 did not lyse the bovine EHEC O157:H7 strain 318 and was non-lytic for the atypical O157:H12 strain B20. Phage 9 lysed the EHEC O157:H7 strains EDL933 and 52, as well as EPEC strains 64 and 65 of the same serotype exhibiting similar EOPs, which is even more interesting as these strains belong to different phage types according to the original study describing them (Tóth et al. 2009). Phage 25 also showed high EOP against EDL933, with notably lower values on other strains. We also tested the phages on strains representing the so-called ‘big six’ serogroups of STEC, as strains belonging to these serogroups make up as high as 83% of non-O157 STEC infections (Gould et al. 2013). One of the phages could lyse the O45 and the O145 strain which were included. However, all phages showed high titre lysis on the O103:H8 and the O121 strain, albeit of an order of magnitude lower than what they showed on the Sakai strain. On the O26 and O111 strains, the EOP was considerably lower. These results show that the phages are capable of lysing pathogenic non-O157 serogroups of *E. coli* as well, albeit their host spectrum does not cover the ‘big six’ serogroups. Although the PCR-based pathotyping showed that the O145 strain carried no *stx* genes, it was included in the EOP testing because of its serogroup membership (Table 2).

As we have noted previously, genomes from members of the same genus differed in only a few SNPs (Sváb et al. 2021). This high level of similarity can only partially be explained by the fact that there were pairs of phages, which originated from the same farm (Table 1), as the five *Dhillonvirus* isolates originated from three independent farms. The same is true for the T4-like phages as they originated from different farms, and the four rV5-like phages obtained were also from three different farms. This uniformity suggests the possibility of a selected range of phages co-habiting domestic cattle with a characteristic *E. coli* population.

Recently, Niu et al (2021) demonstrated that the efficacy of phage cocktails can be affected by synergism or by antagonism between the phage components. In our case, a synergistic tendency was observable when phages were applied as a cocktail, where the 24 h incubation period at 25 °C with MOIs of 10 and 100, was far more effective than when individual phages were applied alone. Nevertheless, the most effective treatment was the application of individual phages for 2 h at 37 °C at a MOI of 100, as this caused a 5 to 6 log reduction in CFU/ml, with no synergistic effect observable in the case of the cocktail. The rates of reduction in all the other cases were lower than those observed in the case of a similar experiment with the rV5-like phage P206 described earlier (Sváb et al. 2018b). Stratakos and Grant observed a 2-log reduction when applying the phages in a MOI of 1000 on cubic pieces of beef (Stratakos and Grant 2018). In similar experiments, Hudson et al. found that MOI values between 1 and 10^4^ result in 1–2 log reduction in the CFU of the O157:H7 strain used (Hudson et al. 2013), although in a later study the authors remark that in the case of surface treatments, MOI is less important than the absolute number of applied phages (Hudson et al. 2015).

For experiments with other foodstuff, using a MOI of 100, similar rates of reduction were observed in the experiment of Sharma et al. (2009) who applied bacteria and phages on the surface of cantaloupe and lettuce. In contrast to these experiments, (Patel et al. 2011) were able to completely eradicate a 10^4^ CFU mixture of five O157 strains from the blade surfaces of a spinach harvester with their applied phage cocktail containing phages at 10^4^ MOI. While it can be argued that the optimal result would be complete eradication, it must be considered that in our experiment and in the cited examples, a high CFU of the target bacteria was used, whereas in a natural setting, a much lower CFU can be expected, therefore very high MOI, and possibly complete eradication is easily achievable with relatively low PFU phage stocks. A further consideration however is that if the bacterial cell density is low, phage density will have to be sufficiently high to ensure that all target cells are infected (reviewed by Hagens and Loessner 2010). Achieving this goal is potentially a more difficult task in non-homogeneous environments (complex foodstuff or live animals) which is a challenge to be considered when designing future experiments and phage biocontrol applications. The comparatively high reduction in the case of using a100-fold MOI at 37 °C is remarkable, but it should be noted that raw beef is rarely stored at this temperature for extended time periods. However, given the fact that the optimal growth temperature of the target bacterium is 37 °C, which in this case seems to be optimal for phage propagation as well, a ‘heat shock’ treatment with a high concentration of phages at this temperature for a limited time can be considered.

## Conclusions

We characterised 11 coliphages of bovine origin, representing the *Tequatrovirus* (T4-like), *Vequintavirus* (rV5-like) and *Dhillonvirus* (HK578-like) genera. Their morphological analysis with TEM confirmed their family-level classification. All showed activity on *E. coli* strains of the O157 serogroup as well as on strains representing the O26, O103, O111 and O121 of the ‘big six’ serogroups of STEC. Detailed phenotypic characterisation of representative phages of the three genera showed that among the phages studied, those belonging to the T4-like and rV5-like genera have a considerable potential for use in treatments aiming at the elimination and biocontrol of *E. coli* O157 in foodstuff.

## Data Availability

Not applicable.
